# Polymeric Fibrous Materials for Procoagulant and Anticoagulant Applications: A Review of Molecular Blood–Material Mechanisms and Strategies

**DOI:** 10.3390/ma19030539

**Published:** 2026-01-29

**Authors:** Marcin H. Kudzin, Monika Sikora, Zdzisława Mrozińska, Jerzy J. Chruściel

**Affiliations:** Łukasiewicz Research Network-Lodz Institute of Technology, 19/27 Marii Skłodowskiej-Curie Street, 90-570 Lodz, Poland; monika.sikora@lit.lukasiewicz.gov.pl (M.S.); zdzislawa.mrozinska@lit.lukasiewicz.gov.pl (Z.M.)

**Keywords:** fiber-forming polymers, polysaccharides, blood coagulation, hemostasis, anticoagulant materials, procoagulant mechanisms, electrospun fibers, surface functionalization, platelet activation, fibrin formation, hemocompatibility, polymer–protein interactions, contact pathway activation, thrombin generation

## Abstract

Fiber-forming polymers are increasingly used to control blood coagulation, either by accelerating the onset of hemostasis or by limiting thrombogenic events in contact with blood. Despite rapid progress in materials engineering, a unified view linking the molecular mechanisms of the coagulation cascade with specific design strategies of procoagulant and anticoagulant polymeric fibers is still missing. In this review, we summarize current knowledge on how natural and synthetic polymers interact with plasma proteins, platelets, and coagulation factors, emphasizing the role of fiber morphology, surface chemistry, charge distribution, and functionalization. Particular attention was paid to systems based on natural polysaccharides (e.g., chitosan, alginate, and cellulose derivatives), as well as synthetic polymers (e.g., PLA, PCL, polyurethanes, and zwitterionic materials). Two possible courses of action were described: their bioactivity may activate the contact pathway and/or support platelet adhesion or their ability to minimize protein adsorption and inhibit thrombin generation. We discuss how metal–polymer coordination, surface immobilization of heparin or nitric oxide donors, and nanoscale texturing modulate coagulation kinetics in opposite directions. Finally, we highlight emerging fiber-based strategies for achieving either rapid hemostasis or long-term hemocompatibility and propose design principles enabling precise tuning of coagulation responses for wound dressings, vascular grafts, and blood-contacting devices. This general compendium of knowledge on blood–material interactions provides a foundation for further design of biomaterials based on fiber-forming polymers and the development of manufacturing processes.

## 1. Introduction

Controlling the balance between clot promotion and clot prevention at a material–blood interface is central to the safe use of polymeric fibers in hemostatic dressings, vascular grafts, catheters, and extracorporeal circuits. When blood meets a foreign surface, proteins adsorb within milliseconds and dictate downstream platelet responses and enzyme activation; this early “conditioning layer” is, therefore, the decisive event that biases a surface toward thrombosis or hemocompatibility [1,2,3]. The properties of the produced polymer fibers, such as fiber diameter, porosity, charge density, degree of hydration, and surface energy, influence the amount, composition, and conformation of adsorbed proteins [2,3,4].

Two conceptual frameworks describe coagulation in this context. The classical “cascade” distinguishes intrinsic (contact activation) and extrinsic (tissue factor, TF) pathways that converge on the common pathway (FX → FXa; prothrombin → thrombin; fibrinogen → fibrin) [5]. The intrinsic arm is initiated when factor XII (FXII) binds to and is activated on negatively charged or otherwise activating surfaces in the presence of high-molecular-weight kininogen (HMWK) and prekallikrein (PK), with reciprocal FXIIa–kallikrein amplification [5]. The extrinsic arm begins when TF-FVIIa complexes activate FIX and FX, providing a rapid route to prothrombinase (FXa-FVa) and a “thrombin burst” that yields fibrin and activates platelets, FXI, FV, and FVIII [5]. Complementing the cascade, the cell-based model reframes coagulation as three overlapping stages—initiation on TF-bearing cells, amplification via platelet activation, and propagation on the activated platelet surface—which better explains spatial regulation, cofactor localization, and why modest early thrombin suffices to trigger explosive thrombin generation later [6,7,8]. In practice, both views are useful: the cascade provides analytic measurands (prothrombin time—PT; kaolin–cephalin time—aPTT; heparin assay—anti-Xa; thrombin–antithrombin III complexes—TAT complexes), whereas the cell-based model clarifies how materials that modulate platelet and cofactor assembly reshape thrombin dynamics [5,6,7,8].

At polymer interfaces, protein adsorption is competitive and dynamic. Early arrivals (e.g., albumin) are often displaced by higher-affinity proteins (e.g., fibrinogen and HMWK)—the Vroman effect—while aging and conformational changes in the adsorbed layer alter ligand exposure to platelet integrins (e.g., GPIIb/IIIa) and contact factors [1,3,9]. These processes underpin two opposing material strategies. Procoagulant fiber mats (often used for topical hemostasis) intentionally promote platelet adhesion/activation and fibrin formation. Here, cationic polysaccharides such as chitosan can electrostatically cluster erythrocytes and platelets and shorten bleeding time; calcium-rich alginate networks and highly wettable cellulose-based fibers concentrate cells and plasma, supporting rapid plug formation [10,11,12]. Electrospun architectures amplify these effects by providing high-surface-area scaffolds with controllable porosity and micro/nanotopography, which facilitate protein deposition and platelet entrapment while enabling drug loading (e.g., thrombin) [13].

Conversely, anticoagulant/antithrombogenic strategies aim to suppress contact activation and platelet triggering on blood-contacting devices. The most established route is heparinization, in which covalently or electrostatically immobilized heparin accelerates antithrombin-mediated inhibition of thrombin and FXa, prolongs plasma recalcification, and reduces device thrombosis without high systemic doses [14,15,16]. A second route uses zwitterionic or ultra-hydrophilic coatings (e.g., phosphorylcholine, sulfobetaine, and carboxybetaine) that form strongly hydrated layers, minimize nonspecific protein adsorption, and dampen platelet adhesion, thereby curbing both contact activation and platelet-driven thrombin generation [8,17,18]. A third, emerging concept does not attempt to eliminate FXII binding; instead, it modulates FXII–surface interactions to prevent its pathogenic activation while maintaining hemostasis, for example, with sheltered positive-charge coatings that bind FXII yet limit reciprocal FXII-PK activation and bradykinin generation [4]. Additional adjuncts, such as nitric oxide-releasing polymers, suppress platelet activation locally and complement antifouling or heparin-based designs [19].

This review situates fiber-forming polymers, including polysaccharides and synthetic fibers, within these mechanistic frameworks. We (1) summarize how polymer charge, hydration, and fiber morphology govern protein corona composition, FXII contact activation, and platelet responses; (2) catalog procoagulant fiber systems for rapid hemostasis versus anticoagulant/antithrombogenic fiber and coating strategies for long-term blood contact; (3) compare trade-offs using a decision matrix grounded in coagulation analytics (PT, aPTT, thrombin generation assays, TAT, and PRT) and platelet assays; and (4) highlight emerging approaches—bioinspired designs, zwitterionic/heparin hybrids, and FXII-targeted surfaces—that promise finer control over the coagulation cascade at polymer–blood interfaces [2,3,4,8,13,14,15,16,17,18,19]. By integrating cascade-centric measurands with cell-based localization principles, we aim to provide material scientists with actionable design rules for procoagulant and anticoagulant fiber-based strategies. Based on the accumulated knowledge, this review situates polymers proposed for fiber-forming processes, primarily polysaccharides (chitosan, alginate, and cellulose derivatives), as well as synthetic polymers (e.g., PLA, PCL, polyurethanes, and zwitterionic materials), within a mechanistic framework. The fiber-forming polymers and fabrication methods mentioned, primarily focused on electrospinning (although we acknowledge that there are several methods), are only examples in this review. We summarize only the main aspects of these main polymers (without focusing on compositional additives) that are important and fundamental to the design of biomaterials and their main blood–material interactions. The collected information constitutes only a foundation of knowledge regarding the biological reactions of biopolymers.

## 2. Fundamentals of the Blood Coagulation Cascade

**Figure 1:**
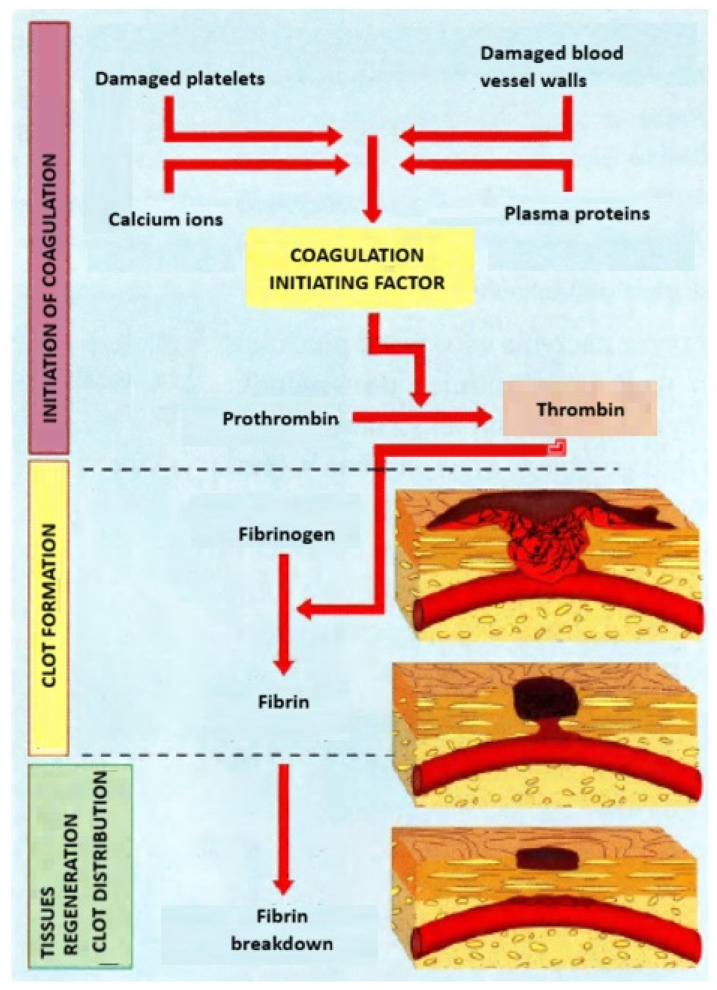
Blood clotting process [3].

Blood coagulation is not a single linear pathway but instead a spatially coordinated network of proteolytic reactions that integrates plasma proteins, platelets, cofactors, and cell membranes to achieve localized fibrin formation. For polymeric fibers used in hemostasis or long-term blood contact, understanding these molecular events is indispensable: the earliest seconds of protein adsorption and platelet engagement determine whether a material accelerates clot formation or successfully suppresses thrombosis [4,5,6,19]. The traditional “intrinsic/extrinsic/common pathway” framework remains valuable for mapping biochemical steps, while the cell-based model explains the spatial and kinetic features of clot formation in vivo [3,4,5,6,20,21]. The schematic process of blood clotting is shown in Figure 1.

### 2.1. Plasma Proteins, Platelets, and the Contact Pathway

Minutes before one observes any macroscopic clot, the decisive molecular interactions occur at the material–blood interface. Plasma proteins adsorb rapidly and competitively, reshaping the surface into a biologically active layer that governs all subsequent cellular events [7,22]. Albumin, fibrinogen, IgG, HMWK, factor XII (FXII), complement factors, and apolipoproteins participate in this early “conditioning” process. The Vroman effect—in which low-affinity, high-abundance proteins are displaced by lower-abundance, high-affinity proteins—modulates not only the composition but also the conformation of adsorbed fibrinogen and HMWK [7,22]. Such conformational transitions expose cryptic binding sites for platelet receptors (GPIIb/IIIa and α5β1) or contact factors, dramatically altering the surface’s procoagulant potential [7,21,23].

The contact pathway is particularly sensitive to material chemistry. FXII binds to surfaces via HMWK and undergoes autoactivation to FXIIa, which reciprocally activates preklikrein to kallikrein, generating a positive feedback loop that accelerates FXII activation and releases the inflammatory mediator bradykinin [20,24]. Kallikrein and FXIIa both bind efficiently to negatively charged or nanostructured interfaces, especially those with high surface area such as electrospun fibers [24,25]. FXIIa then activates FXI → FXIa, which recruits FIX and, in complex with FVIIIa and Ca^2+^, forms the intrinsic tenase complex on activated platelet membranes.

Thus, material features such as surface charge density, hydration layer thickness, nanoscale roughness, and fiber diameter directly influence whether FXII becomes activated or remains quiescent [4,5,20,22]. Platelets simultaneously respond to the adsorbed protein layer: fibrinogen adsorbed in stretched or partially denatured conformations engages GPIIb/IIIa and triggers phosphatidylserine (PS) exposure, while HMWK and kallikrein amplify local contact activation [22,24]. Platelet α-granule secretion releases fibrinogen, FV, FXI, and platelet factor 4 (PF4), further intensifying surface-driven coagulation. These interactions are strongly modulated by hydrophilicity/hydrophobicity gradients in polymeric fibers, the water-binding capacity of polysaccharides, and the presence of multivalent ions such as Ca^2+^ or Cu^2+^ [9,26,27].

### 2.2. Intrinsic, Extrinsic, and Common Pathways

In the intrinsic pathway, FXIIa initiates FXI activation, generating FIXa, which combines with FVIIIa, Ca^2+^, and a negatively charged phospholipid surface to form the intrinsic tense complex. This complex is highly efficient—up to 50-fold more active than its extrinsic counterpart—and drives robust generation of FXa [20,24,25,28]. Because intrinsic pathway activation is closely coupled to surface chemistry, it is particularly relevant for materials science. Surfaces that expose negative charge, acidic polysaccharides, or high-density nanofeatures can accelerate FXII activation; conversely, zwitterionic or hydrated surfaces may attenuate it [9,13,27].

The extrinsic pathway begins when tissue factor (TF) exposed at the injury site forms a complex with FVIIa. TF-FVIIa activates FX and FIX, providing a rapid “ignition signal” that produces small quantities of thrombin even before platelets become fully activated [4,20]. This early thrombin is essential for activating platelets and cofactors FV, FVIII, and FXI. While this pathway is less dependent on the material interface, polymeric fibers applied to wounds (e.g., chitosan, alginate, and cellulose) can modulate extrinsic contributions by concentrating platelets, calcium ions, and TF-bearing microparticles [9,26,27]. Both pathways converge at the common pathway, where FXa and its cofactor FVa assemble the prothrombinase complex on activated platelet surfaces. This yields the thrombin burst required to convert fibrinogen into fibrin, activate FXIII for fibrin crosslinking, and stabilize the clot [20,21,25].

Thrombin also activates thrombin fibrinolysis inhibitor (TAFI), which modifies fibrin structure and slows fibrinolysis, adding another layer of regulation [21]. Materials that accelerate platelet activation or concentrate fibrinogen strengthen the thrombin burst; anticoagulant materials such as heparinized fibers inhibit FXa and thrombin directly, dampening prothrombinase activity [13,14].

### 2.3. Cellular Model and Spatial Regulation of Thrombin on Fibrous Materials

The cell-based model clarifies aspects of coagulation that cannot be captured by the cascade alone. In the initiation phase, TF-bearing cells generate a small amount of thrombin sufficient to activate platelets and cofactors. During amplification, thrombin-activated platelets expose PS-rich domains and recruit FXI, FV, FVIII, and FIX, forming a surface competent for assembling tenase and prothrombinase complexes [4,5,6,20,21].

In the propagation phase, these complexes generate a surge of thrombin that drives fibrin formation [20,21]. Spatial regulation is crucial: coagulation occurs almost exclusively on cellular or cell-like surfaces. Polymeric fibers—by virtue of their large surface area, tunable hydrophilicity, and modifiable charge—can either mimic platelet surfaces and support full assembly of tenase/prothrombinase (procoagulant materials) or present non-adhesive, protein-repellent interfaces (anticoagulant materials) [9,13,26,27]. Heparinized fibers locally inhibit FXa and thrombin without suppressing upstream contact activation [13,14]. Zwitterionic coatings disrupt protein adsorption and limit both platelet adhesion and FXII contact activation [9,13,27]. Nitric oxide-releasing fibers suppress platelet activation, reducing the probability of thrombin propagation even when small amounts of thrombin are generated [29].

### 2.4. Analytical Tests Characterizing the Interaction of Hemostatic Materials with Blood

A meaningful interpretation of material–coagulation interactions requires assays aligned with the specific mechanism being studied. PT (prothrombin time) reflects extrinsic/common pathway activity and is influenced by TF-FVIIa-FX activation. aPTT (activated partial thromboplastin time) reflects intrinsic/common pathways and is particularly sensitive to FXII and FXI activation; it is therefore widely used for polymer surfaces suspected of triggering contact activation [3,20,25].

Thrombin generation assays (TGAs) provide kinetic information (lag time, peak thrombin, and endogenous thrombin potential) that distinguishes initiation defects from propagation defects, which is critical when comparing procoagulant vs. anticoagulant surfaces [23,24,25,29]. TAT complexes quantify in-plasma thrombin production and are widely used in ex vivo loop systems for dialyzers, catheters, and vascular grafts [13,14].

Platelet assays (adhesion/spreading, P-selectin exposure, integrin activation via PAC-1 binding, and GPIIb/IIIa clustering) reveal the material’s effect on the amplification stage [4,6,7,21]. Fibrin formation kinetics can be monitored via turbidity, confocal microscopy, or SEM/optical analysis of fiber networks.

For topical hemostats (chitosan, alginate, and cellulose), standardized bleeding models (rat liver, rabbit ear, and porcine liver) quantify time to hemostasis and total blood loss [9,26,27]. For blood-contacting materials, hemolysis, complement activation, and platelet consumption assays ensure global biocompatibility [13,14]. Matching assays to intended use prevents false conclusions: for example, a material may show prolonged aPTT in vitro but still support adequate hemostasis in vivo because it acts mainly on propagation rather than initiation. Conversely, a fiber may appear hemocompatible under static conditions but trigger FXII under flow due to altered protein adsorption dynamics [7,22,23,24].

## 3. Interactions of Polymers with Proteins Regulating Blood Coagulation

Polymers interacting with blood are rapidly coated by a dynamic and spatially heterogeneous layer of adsorbed plasma proteins. This “conditioning layer” becomes the true biological interface, regardless of the underlying chemistry of the material, and is, therefore, the key determinant of coagulation outcomes, platelet behavior, and long-term hemo-compatibility [2,4,30]. In fiber-forming polymers, high surface-area-to-volume ratios, nano-/microscale curvature, and tunable chemistries intensify these early events, creating interfacial microenvironments that either accelerate or suppress coagulation. Understanding how this protein corona forms, evolves, and signals to coagulation proteins and platelets is fundamental for designing both prohemostatic dressings and anticoagulant blood-contacting devices.

Immediately after blood contacts a polymeric surface, protein adsorption occurs on a millisecond timescale. Albumin, fibrinogen, HMWK, FXII, complement proteins (C3/C4), apolipoproteins, and IgG compete for surface sites [21,31]. Because each protein has distinct affinities, hydration requirements, and conformational stability, the composition of the early corona is highly transient. The Vroman effect describes the displacement of initially adsorbed, abundant proteins by less abundant but higher-affinity proteins such as fibrinogen and HMWK [7,22,32].

One decisive feature is adsorption-induced protein deformation. Fibrinogen can adopt extended conformations on hydrophobic or hydrogen-bonding surfaces, exposing γ-chain dodecapeptide (H12) sequences that strongly engage platelet GPIIb/IIIa receptors and initiate platelet adhesion [7,26]. Similarly, HMWK, once adsorbed, can orient its do-main 5 toward the surface, presenting binding motifs that facilitate FXII docking [20]. The degree of unfolding is strongly influenced by local curvature; submicron fibers create high curvature radii that favor partial denaturation and increase the probability of cryptic epitope exposure compared with planar films [33].

On hydrophilic, strongly hydrated surfaces, the opposite occurs: proteins retain native-like conformations, exhibit reduced residence times, and show diminished ability to form stable adhesive domains. This weaker corona correlates with lower platelet activation and reduced FXII binding, underpinning the antithrombogenicity of zwitterionic and ultra-hydrophilic coatings [8,16].

### 3.1. The Role of Surface Charge, Hydration Force, and Zeta Potential Between Polymers and Plasma Proteins

Surface charge modulates electrostatic attraction between polymers and plasma proteins. Cationic polymers, such as protonated chitosan, attract negatively charged domains of fibrinogen and albumin and can directly bind negatively charged platelets. This electrostatic clustering drives platelet activation, granule release, and rapid thrombin generation, making cationic fibers effective for topical hemostasis [9].

Conversely, anionic surfaces (e.g., alginate and carboxymethylcellulose) attract cations such as Ca^2+^, which can locally elevate coagulation cofactor concentrations and promote fibrin polymerization. However, at high anionic densities, they can also stabilize FXII/HMWK complexes and inadvertently activate the contact pathway [20,34].

Zwitterionic surfaces (phosphorylcholine, sulfobetaine, and carboxybetaine) exhibit tightly bound hydration shells that form a physical and energetic barrier to protein adsorption [8,16]. These hydration barriers arise from strong dipole interactions that structure interfacial water molecules, resisting protein displacement and suppressing unfolding [16]. As a result, coronas formed on zwitterionic surfaces are sparse, weakly interacting, quickly exchanging, and poor at enabling platelet docking or FXII anchoring.

Hydration also affects zeta potential, which influences the distribution of ions and proteins within the electrical double layer. Fibers with strongly negative zeta potential may preferentially recruit HMWK and FXII, whereas zwitterionic materials maintain low surface potentials that disfavor binding of both platelets and contact factors [8,16].

**Figure 2:**
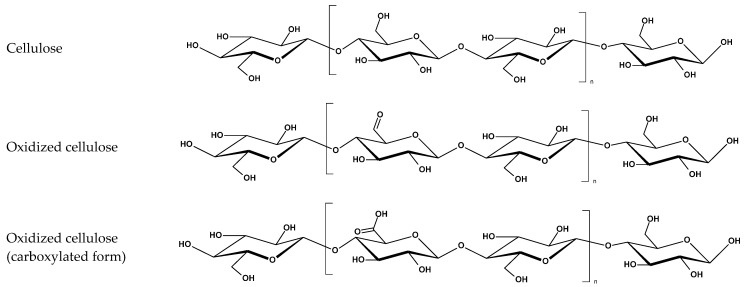

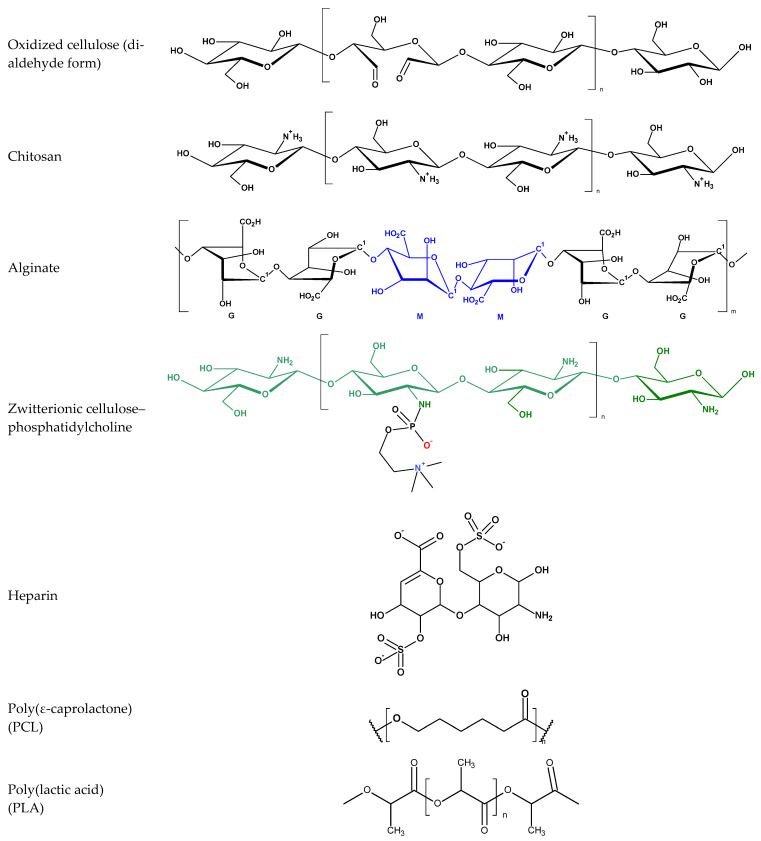
Chemical structures of polysaccharides and synthetic polyesters relevant to coagulation-modulating materials. Key functional groups associated with surface charge, hydration behavior, and interactions with contact-activation proteins are highlighted [8,9,20].

To clarify how specific functional groups and charge-distribution motifs contribute to the electrostatic interactions, hydration-mediated effects, and protein-binding patterns described in the previous section, the key chemical structures of representative procoagulant and anticoagulant polymers are summarized in Figure 2, providing a molecular framework for interpreting their divergent behaviors at the blood–material interface.

### 3.2. Nanoscale Fiber Topography, Diameter, Porosity, and Mechanical Strength

Topographical material features shape how proteins pack and how platelets sense the surface. Submicron fibers create high curvature, promoting selective adsorption of flexible proteins such as fibrinogen while disfavoring structured globulins [13,33]. Porosity and capillarity determine plasma transport within fibrous scaffolds; high porosity accelerates wicking and facilitates local concentration of proteins, platelets, and red blood cells, features that are beneficial for rapid hemostasis but problematic for long-term device contact [14].

Mechanical characteristics compliance matters as well. Platelets preferentially adhere to stiffer surfaces; compliant or gel-like coatings resist focal adhesion formation, reducing spreading and integrin activation. Soft coatings, especially hydrated zwitterionic hydrogels, therefore reduce platelet activation even when some corona forms [8,16,23].

### 3.3. Contact Activation at Polymer Interfaces: FXII/HMWK/Prekallikrein Microenvironment

Contact activation is a surface-driven event, and its efficiency depends on the ability of the corona to recruit FXII and orient it for autoactivation. HMWK plays a pivotal role: it binds surfaces via its domain 3 and presents FXII-binding domains that facilitate FXII → FXIIa conversion [20,35]. Surfaces rich in carboxyl or sulfonate groups stabilize FXII/HMWK complexes, while hydrated matrices (oxidized cellulose and alginates) support both HMWK accumulation and FXII docking [20,34].

Once FXIIa appears, it activates prekallikrein to kallikrein, which, in turn, enhances FXII autoactivation and releases bradykinin, linking coagulation with inflammation and vasodilation [35]. These feedback loops explain why certain polymers appear disproportionately procoagulant in aPTT tests even if they lack prothrombotic behavior in vivo [20,35].

**Figure 3:**
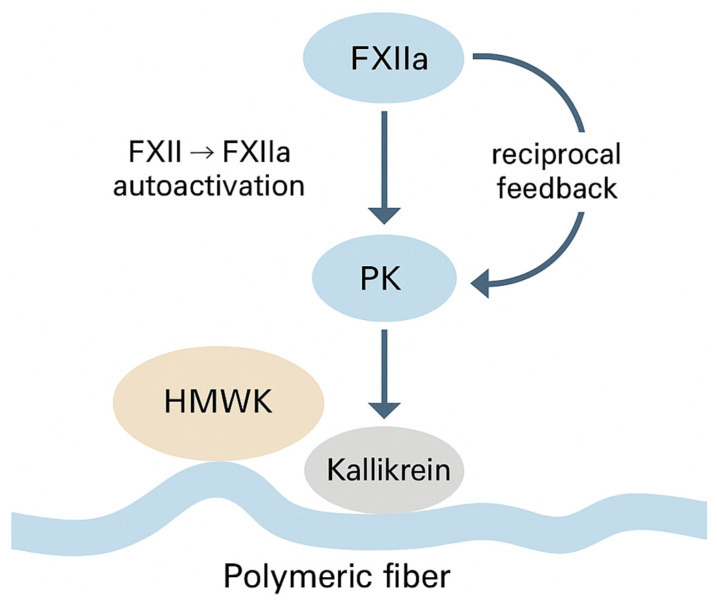
Surface-driven contact activation pathway on polymeric fibers. Adsorbed HMWK anchors FXII to the material surface, enabling FXII → FXIIa autoactivation and subsequent conversion of prekallikrein (PK) to kallikrein. Kallikrein amplifies FXII activation through a reciprocal feedback loop, collectively accelerating intrinsic pathway initiation as described in polymer–blood interaction studies [20,24,35].

The sequence of interfacial molecular events governing FXII/HMWK binding, FXII autoactivation, and the reciprocal FXIIa–prekallikrein amplification loop on charged polymeric fibers is summarized in Figure 3, integrating mechanistic insights described in recent studies on contact-mediated coagulation activation [20,24,35].

Anticoagulant surfaces intervene at distinct points. Heparin-functionalized fibers inhibit FXa and thrombin, preventing amplification even if contact activation occurs upstream [36,37]. Zwitterionic coatings reduce the probability that FXII/HMWK complexes form [8,16]. NO-releasing coatings suppress platelet activation locally [38].

### 3.4. Platelet Adhesion, Activation, Spreading, and Membrane Support for Enzymatic Complexes on Fibrous Materials

Platelets integrate biochemical and biomechanical cues from the corona. Adsorbed fibrinogen (especially in extended conformations) engages GPIIb/IIIa, triggering calcium influx and α-granule secretion. P-selectin exposure increases adhesion to leukocytes and amplifies inflammatory signaling [7,21,26]. Platelets then flip phosphatidylserine (PS) to the outer leaflet, creating a catalytic platform for assembling intrinsic tenase (FIXa-FVIIIa) and prothrombinase (FXa-FVa) [30,31].

Procoagulant polymers—especially those with cationic or rough fibrous surfaces—accelerate these processes by providing anchoring sites and promoting formation of fibrinogen clusters with high GPIIb/IIIa affinity [9,14,33].

Anticoagulant fibers disrupt this cycle: zwitterionic hydration layers prevent stable integrin–ligand binding; NO-releasing surfaces interfere with platelet cytoskeletal reorganization; and hydrated, compliant hydrogels reduce mechanotransduction needed for integrin clustering [8,16,23,38].

### 3.5. Mapping Interfacial Phenomena to Coagulation Metrics of Fibrous Materials

Accurate mechanistic interpretation requires testing aligned with the affected pathway. aPTT detects intrinsic/contact activation (FXII/FXI) and responds strongly to surfaces that recruit HMWK/FXII [20,35]. Thrombin generation (TG) assays dissect initiation vs. propagation and correlate with platelet activation [33,36]. TAT complexes reflect cumulative thrombin formed under dynamic conditions [37]. Platelet activation panels (P-selectin and PAC-1 binding) identify materials triggering amplification [7,21,26]. Bleeding models evaluate hemostatic performance of chitosan-, alginate-, and cellulose-based fibers [9,13,14,34]. Complement activation/hemolysis tests reveal inflammatory responses for long-term blood-contact devices [37,38].

The interfacial behavior of polymer–protein systems can be rationalized as a set of mechanistic levers—charge, hydration, topography, mechanical stiffness, and functionalization—that together shape protein corona architecture and platelet coagulation dynamics. Designing procoagulant fibers involves enhancing protein capture, platelet adhesion, and FXII engagement, whereas designing anticoagulant or antithrombogenic fibers requires minimizing protein adsorption, inhibiting FXa/thrombin, or suppressing platelet activation. To clarify how specific material features govern coagulation behavior, the key physicochemical parameters of fiber-forming polymers and their mechanistic influence on blood coagulation pathways are summarized in Table 1.

### 3.6. The Influence of Hemorheology on Coagulation Process

Hemorheology is the study of blood flow and deformation, which has a fundamental impact on the clotting process, particularly in contact with biomaterials. Key hemorheological phenomena determine the rate and location of clot formation through parameters such as viscosity, shear stress, and mass transport of blood components [40,41].

Blood flow-induced stress is the primary mechanical factor regulating platelet activity. High shear stress (high shear) occurs in constricted vessels and at the edges of implants. This leads to platelet activation and aggregation dependent on von Willebrand factor (vWF) [40]. Extremely high shear stresses cause mechanical damage to red blood cells (hemolysis), which releases ADP and further stimulates coagulation. Low shear stress, on the other hand, promotes cell sedimentation and activation of the intrinsic pathway (contact phase). In areas of blood stasis (e.g., behind valves, in “dead zones” of flow), clots rich in fibrin and red blood cells (red clots) form [41,42].

Blood viscosity is not constant (non-Newtonian fluid) and depends on the hematocrit, aggregation, and elasticity of red blood cells, as well as the plasma proteins themselves [43]. A higher hematocrit increases viscosity and pushes platelets toward the vessel walls (platelet marginalization), facilitating their contact with the biomaterial surface. Furthermore, the ability of blood cells to form deformations, known as “rolls,” increases viscosity at low flow rates, promoting prothrombotic blood stasis [43,44]. High fibrinogen concentrations can also directly increase plasma viscosity, facilitating the formation of bridges between platelets [41].

Hemorheology controls the delivery of coagulation substrates to the surface, such as the delivery of factors and the flushing of inhibitors. Laminar and turbulent flow determine how quickly coagulation factors and platelets reach the site of activation on the polymer surface. Rapid flow can inhibit coagulation by diluting and flushing out active enzymes (e.g., thrombin) and natural anticoagulants [43].

In dynamic (flow) conditions, clots have a different structure than they do at rest. On flat biomaterial surfaces, hemorheology forces the formation of structures elongated in the flow direction, which can lead to their sudden detachment and embolism [44]. Understanding these processes is currently crucial for the design of hemocompatible coatings, which must be tested under dynamic flow conditions, not just in static tests, to truly assess their safety [44,45].

Current biomaterial design strategies focus on actively controlling hemorheology to prevent flow-induced activation of the coagulation cascade. A key goal is to create a surface that “deceives” the blood by minimizing mechanical and chemical prothrombotic stimuli. The choice of strategy depends on the anticipated flow regime (arterial–high pressure/high shear; venous–low pressure/risk of stasis). Combining several methods is standard (e.g., smooth geometry + zwitterion coating) [45,46].

One of the main biomaterial strategies that address hemorheology is slippery liquid-infused porous surfaces. Blood slides across a fluid layer rather than touching a rigid polymer [47]. This eliminates protein adhesion and drastically reduces local shear stress at the interface, preventing platelet activation even at slow flow rates. Designing the device geometry is also crucial. Polymer fibers are aligned with the flow vector, minimizing hydrodynamic resistance and promoting orderly endothelial cell deposition. Biomaterials are designed to actively modify local blood rheology and chemistry [47,48]. Polymer coatings (e.g., polyurethanes with NO donors) mimic natural endothelium. NO not only inhibits platelet adhesion but also locally dilates vessels, reducing shear stress. Permanently binding heparin to a flat surface (dialysis membrane) allows for thrombin inactivation directly at the wall before it can affect local plasma viscosity [43]. The use of zwitterionic polymers or PEGylation (coating with a layer of polyethylene glycol) creates a dense “cloud” of water molecules on the material’s surface. Blood cells (erythrocytes and platelets) are blinded to the polymer surface, preventing marginalization and friction against the walls and maintaining the natural blood viscosity near the implant [48,49]. Recent research indicates that substrate stiffness also influences clotting. By mimicking the elastic nature of natural vascular tissue, soft hydrogels can reduce the mechanical activation of platelets that occurs on hard metal or plastic surfaces under the influence of pulsatile blood flow [49].

## 4. Procoagulant Fiber-Forming Polymers

Procoagulant fiber-forming polymers are engineered to accelerate the earliest events of the coagulation cascade—protein adsorption, platelet recruitment, contact activation, and the assembly of enzymatic complexes—ultimately driving rapid thrombin generation and stable fibrin formation. Compared with films or bulk hydrogels, fibrous materials provide exceptionally high surface area, well-defined porosity, and controllable topography, which together yield strong capillary action, rapid fluid uptake, and efficient concentration of coagulation components at the blood–material interface [8,27]. These features make fiber-based architectures uniquely suited for topical hemostasis, trauma care, surgical applications, and wound environments where the speed of clot formation determines performance [4,39].

Below, we dissect the major classes of procoagulant fiber-forming polymers—natural polysaccharides, synthetic fibers, and ion-modified composites—and explain how their physicochemical properties modulate each mechanistic checkpoint of coagulation.

### 4.1. Natural Polysaccharides with Intrinsic Hemostatic Potential

#### 4.1.1. Chitosan: A Cationic Polymer with Strong Platelet-Interactive Properties

Chitosan remains one of the most widely used prohemostatic biopolymers. Its primary amine groups (pKa ≈ 6.3–6.5) are protonated at physiological pH, yielding a persistent positive charge that binds electrostatically to negatively charged erythrocytes, platelets, fibrinogen, and albumin [9]. As a result, chitosan fibers effectively cluster platelets, promote α-granule release, and facilitate early phosphatidylserine (PS) exposure, critical steps for assembling intrinsic tenase and prothrombinase complexes in the cell-based model of hemostasis [8,27].

Electrospun chitosan or chitosan-blended fibers exhibit strong capillary uptake and rapidly concentrate clotting factors at the wound site. Adsorption of fibrinogen in partially denatured conformations lowers the activation threshold for GPIIb/IIIa-mediated platelet adhesion and accelerates generation of thrombin [4,50]. In vitro assays typically show shortened aPTT, reduced thrombin generation (TG) lag time, and increased peak thrombin for chitosan matrices, while animal bleeding models consistently demonstrate reduced time to hemostasis and decreased blood loss [9,50,51].

Chitosan’s positive charge also supports cohesion of the forming clot, acting as a biological glue that binds the nascent fibrin network. Its intrinsic antibacterial properties offer clinical advantages in contaminated or trauma-associated wounds [9,51].

#### 4.1.2. Alginate: An Anionic Polysaccharide Enabling Ca^2+^-Mediated Gelation and Fibrin Reinforcement

Alginate fibers form hydrophilic, highly absorbent gels in the presence of Ca^2+^, making them particularly effective for wounds with significant bleeding and exudate [52]. Their hemostatic action emerges from several synergistic mechanisms: capillary concentration of plasma proteins and cells within the fibrous mesh, creating a microenvironment rich in fibrinogen and platelets; the release of Ca^2+^, which accelerates the activation of factor X, prothrombin-to-thrombin conversion, and fibrin polymerization [52,53]; and moderate stabilization of FXII/HMWK complexes on highly anionic surfaces, which can facilitate contact pathway activation, useful in topical hemostats but undesirable in long-term blood-contact devices [26].

Porous alginate fibers promote rapid dehydration of wound surfaces, increasing local protein concentration and reducing the distance required for platelet–platelet and platelet–fibrin interactions. Together with Ca^2+^-driven fibrin reinforcement, alginate mats produce structurally robust clots capable of withstanding mechanical stress [52,53].

#### 4.1.3. Cellulose-Based Fibers

Cellulose and its derivatives, especially oxidized regenerated cellulose (ORC), display rapid absorbency, high surface area, and controlled microfibrillar architecture that together enhance clot adherence and structural stability [39,54]. ORC introduces carboxyl groups that (1) increase water uptake, (2) promote strong fibrinogen adsorption, and (3) can stimulate FXII/HMWK localization, mildly enhancing intrinsic pathway activation [4,26,54].

The capillary action of cellulose fibers creates swift plasma wicking, concentrating platelets and coagulation proteins directly within the fiber bed. This mimics the functional role of activated platelet membranes in the propagation phase of coagulation, facilitating the formation of dense fibrin structures and improving clot anchoring to the wound bed [8,27,39].

**Figure 4:**
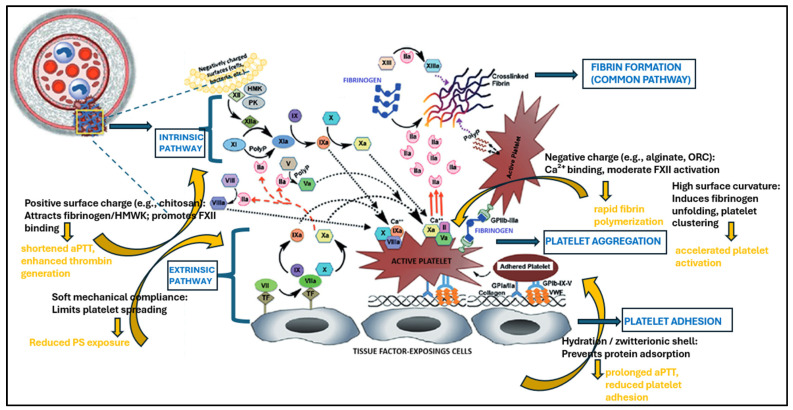
Schematic diagram of the complex mechanism of vascular hemostasis. Vessel damage can lead to endothelial activation and denudation, and the secretion and deposition of von Willebrand factor (vWF), collagen, and tissue factor (TF)-positive cells occurs at the site of injury; summary of polymer physicochemical parameters influencing blood coagulation pathways [4,9,39,51,52,53,54].

In summary, for polysaccharide-based fibers, the interplay of charge (positive for chitosan; negative for alginate and oxidized cellulose), hydration capacity, and structural porosity governs protein corona formation and platelet activation, resulting in shortened aPTT and thrombin generation lag times, as well as reduced bleeding duration in vivo, as shown in Figure 4 [4,9,39,51,52,53,54].

### 4.2. Synthetic Polymers Engineered for Rapid Hemostasis

Synthetic polymers such as poly(lactic acid) (PLA), polycaprolactone (PCL), polyurethane (PU), and PEG (polyethylene glycol)-modified copolymers gain strong procoagulant activity after electrospinning, which imparts submicron fiber diameters with high curvature; interconnected porosity with tunable tortuosity; strong wicking capability and rapid fluid transport; and microenvironments that enhance fibrinogen adsorption and unfolding [4,50,55].

Curvature-induced fibrinogen deformation exposes platelet-binding motifs (γ-chain H12), increases integrin clustering, and accelerates platelet activation [4,50]. When blended with chitosan or coated with calcium-enriched layers, these effects become more pronounced, producing significant increases in peak thrombin and rapid propagation in TG assays [50,55].

PLA/PCL fibers also promote formation of a dense fibrin cap due to their physical structure: platelet aggregates become mechanically interlocked between fibers, stabilizing nascent thrombi even under shear [55]. The combination of topography, rapid dehydration, and controlled hydrophobicity makes synthetic fibers versatile platforms for tuning procoagulant performance [4,50,55].

### 4.3. Metal–Polymer Coordination Systems: Ca^2+^, Zn^2+^, and Cu^2+^ as Accelerators of Coagulation Microenvironments

Incorporating metal ions such as Ca^2+^, Zn^2+^, and Cu^2+^ into fibers improves hemostasis through biochemical and structural mechanisms. Ca^2+^ is directly required for the assembly of intrinsic tenase (FIXa-FVIIIa) and prothrombinase (FXa-FVa), enhances fibrin polymerization and clot thickening, and also forms crosslinks with alginate, improving capillary flow and mechanical stability [26,52]. Zn^2+^ and Cu^2+^ are ions that coordinate with protein side chains, altering adsorption patterns and promoting platelet adhesion. They modulate hydration layers, shifting the composition of the protein corona toward fibrinogen- and HMWK-rich profiles [8,26,56]. Zn^2+^ and Cu^2+^ ions often add antimicrobial activity critical for trauma environments [56].

Metal-enriched fibers typically exhibit shorter aPTT, higher TG peak thrombin, and improved performance in in vivo bleeding models [52,53,56]. However, excessive metal content risks cytotoxicity and inflammatory activation, making careful stoichiometric control essential [56].

The relationship between chemical structure, surface charge, and biological function determines whether a fiber-forming polymer promotes coagulation or maintains hemocompatibility. Table 2 summarizes the dominant functional groups, electrostatic properties, and corresponding effects on blood coagulation for representative natural and synthetic polymers discussed in this review.

### 4.4. Crosslinking Strategies, Surface Charge Tuning, and Microenvironment Engineering

The crosslinking density of fiber networks modulates mechanical stiffness, an important factor for platelet mechanotransduction. Slightly stiffer mats support platelet spreading and PS exposure, enhancing propagation, whereas overly soft hydrogels reduce integrin clustering and may slow clot formation [8,27,47]. Chemical or ionic crosslinking also changes water retention, affecting corona dynamics, and surface charge density, influencing FXII/HMWK binding and pore architecture and determining the depth of plasma penetration and fibrin formation [26,27,57]. Cationic modification (e.g., amination) can significantly increase platelet adhesion and fibrinogen unfolding but must be balanced to avoid hemolysis or excessive inflammation [9,56,57].

### 4.5. Electrospun Prohemostatic Composites: Synergistic Fiber Systems

Electrospinning allows precise control of fiber diameter, alignment, porosity, and multilayer architecture. Combining synthetic polymers (PLA/PCL/PU) with bioactive polysaccharides (chitosan and alginate) creates hybrid hemostatic systems that unify capillary wicking (synthetic layer), strong platelet adhesion (cationic polysaccharide layer), and Ca^2+^-mediated fibrin reinforcement (alginate or metal-containing layer) [9,50,51,52,53,55,56]. These materials exhibit shortened TG lag time, elevated thrombin burst, accelerated clot formation in ex vivo perfusion tests, and significantly faster hemostasis in animal bleeding models. Multilayer constructs also manage exudate effectively and promote wound healing by maintaining an optimal moisture balance [9,50,51,52,53,55].

The correct fibrillar (fibrous) geometry of biomedical polymers is crucial for their hemocompatibility, meaning their ability to come into contact with blood without causing undesirable reactions such as hemolysis or coagulation activation. The main geometric parameters influencing thrombogenic properties are fiber diameter and nanostructure. Nanoscale fibers, such as those obtained by electrospinning, can mimic the natural extracellular matrix (ECM). A sufficiently small fiber diameter and their dense packing can limit the adsorption of plasma proteins (e.g., fibrinogen), which is the first step in the clot formation process [33,38,55]. Porosity and orientation, i.e., the spatial structure of fibrillar scaffolds, are also crucial. An open, porous structure promotes reendothelialization (endothelial cell growth). The formation of a continuous endothelial layer is the most effective natural way to prevent thrombosis. It is crucial that the fiber geometry minimizes turbulence and flow instabilities, which mechanically contribute to platelet activation [50,57]. Excessive fiber surface roughness (often defined as the Ra parameter) increases the risk of platelet adhesion and activation. Quantitative geometry assessment using fractal dimensions allows for precise classification of materials in terms of their thrombogenic potential (TP). Optimal fibrillar geometry should strive for biomimeticity (mimicking natural vessels) while maintaining very low roughness and a high ability to rapidly coat the patient’s own endothelium [39,52].

Hemostatic processes on biomaterials with flat geometry (membranes, films, and hydrogels) are initiated by physicochemical interactions at the material–blood interface. Unlike porous structures, where mechanical trapping is crucial, molecular phenomena such as protein adsorption and the Vroman effect, contact phase activation (intrinsic pathway), primary hemostasis (platelet adhesion and aggregation), and secondary hemostasis (fibrin formation) are crucial on flat surfaces [5,8,9,57].

On flat materials, the composition of the surface layer depends on surface energy and hydrophilicity. In the case of hydrogels, high water content and swelling allow for the concentration of coagulation factors and red blood cells (erythrocytes) at the wound site through selective absorption of the liquid portion of plasma [52]. Some hydrogels (e.g., chitosan) possess a natural positive charge that electrostatically attracts negatively charged erythrocytes, accelerating clotting. Membranes act as a physical barrier, promoting a local increase in thrombin and fibrin concentration and preventing their dilution by blood flow [50,55].

### 4.6. Translational Considerations for Procoagulant Fiber Design

Because each fiber property—charge, topography, hydration, and stiffness—modulates a specific aspect of the coagulation cascade, rational design requires mapping material characteristics to mechanistic control points. Contact activation (FXII), modulated by surface charge, hydrogen bonding, and affinity for HMWK, appears to be an important control point [8,26,27]. Platelet adhesion and activation are modulated by fibrinogen conformation (roughness and curvature) [4,40,45]. The propagation stage (tenase/prothrombinase) is influenced by capillary concentration, PS exposure, and Ca^2+^ availability [8,26,27,52,53]. During fibrin network formation, porosity, permeation rate, and fiber interlocking are key factors [4,39,52,55].

For clinical translation, in vitro metrics (aPTT, TG, platelet activation assays, and TAT) must be paired with animal bleeding models, which provide realistic assessments of time to hemostasis and blood loss [9,50,51,52,53,54,55,56]. For metal-based composites, biocompatibility, cytotoxicity, and inflammatory responses require careful evaluation to maintain safety [56,57]. Natural polymers (e.g., collagen) mimic the extracellular matrix (ECM), facilitating cell adhesion. Synthetic polymers require additional functionalization. Natural materials carry the risk of immunogenicity or the transmission of zoonotic pathogens. Synthetic materials are safer and more reproducible in this regard. The current trend is to create hybrid composites that combine the durability of synthetic polymers with the bioactivity of natural ones [56,57]. Table 3 presents a comparison of natural polymers (such as collagen, chitosan, and alginate) and synthetic polymers (e.g., PLA, PGA, PCL, and polyurethanes) in key biomedical aspects.

## 5. Strategies for Designing Anticoagulant and Antithrombogenic Fiber-Based Materials

Designing fiber-based materials that maintain blood compatibility over extended contact periods represents one of the most demanding challenges in biomaterials science. While prohemostatic fibers aim to accelerate clotting in localized wound environments, anticoagulant and antithrombogenic fibers must perform the opposite function: to prevent surface-induced thrombosis, uncontrolled thrombin generation, and platelet aggregation while remaining cytocompatible and mechanically durable. Such systems are critical in vascular grafts, stents, extracorporeal circuits, catheters, and dialysis membranes, where long-term exposure to circulating blood demands exquisite balance between chemical inertness and controlled biological signaling [65,66,67,68,69,70].

Coagulation control in these contexts is not achieved by a single inhibitory mechanism but rather through multi-level interference with the three overlapping stages of the cell-based model: (1) initiation-dominated by protein adsorption and contact activation; (2) amplification-driven by platelet activation and PS exposure; and (3) propagation-sustained by enzyme complexes and thrombin feedback [4,65,66]. The most effective fiber-based anticoagulant systems target at least one of these checkpoints while maintaining endothelial-mimetic hydration and minimal immune activation.

### 5.1. Heparin-Functionalized Fibers: Localized Suppression of Enzymatic Propagation

Heparin immobilization remains the gold standard in hemocompatible surface engineering. Its highly sulfated polysaccharide chains bind antithrombin III (ATIII), accelerating the inhibition of FXa and thrombin (FIIa) by up to 1000-fold. When tethered to fiber surfaces, this process forms a localized catalytic interface that deactivates clotting enzymes as they approach the material, thus preventing propagation of thrombin generation [68,69,71]. Many immobilization strategies have been developed, including covalent conjugation (via carbodiimide or epoxide chemistry), which provides long-term stability under shear but may reduce heparin mobility and thus ATIII availability.

The layer-by-layer (LbL) assembly strategy creates dense polyelectrolyte multilayers with tunable heparin charge; however, weak electrostatic interactions may cause partial desorption under flow influence. In contrast, plasma grafting allows conformal deposition on complex fibrous geometries while maintaining high permeability [68,69,71].

In vitro assays reveal prolonged aPTT, decreased thrombin generation (TG) peak and endogenous thrombin potential (ETP), and reduced TAT complex accumulation in dynamic perfusion systems [66,67,69,72]. Scanning electron microscopy (SEM) and synchrotron imaging confirm minimal fibrin deposition and platelet adhesion on heparinized fibers compared with unmodified controls. Mechanistically, while heparin does not fully block contact activation (FXIIa generation), it truncates the propagation phase by suppressing downstream enzymatic amplification [4,65,66].

Long-term performance depends on heparin density and orientation. Too low surface coverage yields incomplete enzyme neutralization, while excessive loading can alter mechanical integrity or delay endothelialization. Optimal heparin densities (on the order of 1–3 µg/cm^2^ or a few tens of pmol/cm^2^ of active ATIII-binding sites) produce maximal ATIII-mediated inhibition with minimal surface instability [68,69,71,73]. Clinically, heparinized hollow fibers and vascular grafts exhibit markedly reduced thrombus formation and longer operational lifetimes compared to untreated analogs [68,69,73].

### 5.2. Nitric Oxide (NO)-Releasing Coatings: Biomimetic Signaling

Nitric oxide (NO) is an endogenous gaseous messenger released by endothelial cells to maintain platelet quiescence, inhibit leukocyte adhesion, and regulate smooth muscle tone. Reproducing this antiplatelet signaling on fiber-based materials has become an advanced strategy for long-term hemocompatibility [74,75,76].

Modern NO-releasing coatings combine NO donors (diazeniumdiolates, S-nitrosothiols, or NONOates) with hydrophilic polymer matrices that modulate release kinetics. The introduction of zwitterionic or amphiphilic block copolymers as outer layers improves both storage stability and control of flux, maintaining physiological NO levels (≈0.5–4 × 10^−10^ mol cm^−2^ min^−1^) over days or weeks [74,75]. This localized NO release suppresses platelet integrin activation (GPIIb/IIIa), reduces P-selectin exposure, and limits phosphatidylserine (PS) translocation, thus directly hindering the amplification phase of the cell-based coagulation model [4,65,66,74].

For fibrous constructs, NO donors can be embedded within the polymer matrix or covalently attached to fiber surfaces, enabling spatially controlled, continuous release. Electrospun NO-releasing mats have demonstrated >80% reduction in platelet adhesion and significant attenuation of thrombin generation compared with untreated controls [74,75,76]. Unlike systemic NO donors, local delivery minimizes hypotension risks. However, flux optimization is essential: excessive NO accelerates oxidative degradation of the polymer and may generate cytotoxic species (peroxynitrite), while insufficient release fails to prevent platelet activation [75,76].

Recent hybrid systems combine NO-releasing layers with zwitterionic armor, producing dual benefits: antifouling (adsorption resistance) and active platelet suppression [74,76,77]. Such multifunctional designs achieve near-endothelial performance under flow, with minimal fibrin or complement activation.

### 5.3. Zwitterionic and Ultra-Hydrophilic Interfaces: Preventing Protein Adsorption and Contact Activation

Zwitterionic materials, such as phosphorylcholine (PC)-, sulfobetaine (SB)-, and carboxybetaine (CB)-based polymers, create highly stable hydration shells through strong ionic dipoles that attract and structure water molecules [70,78,79]. This hydration layer acts as an energetic barrier, reducing nonspecific adsorption of plasma proteins by more than 90% compared with PEGylated surfaces, even at physiological salt concentrations [78,79].

In the context of fiber systems, zwitterionic coatings or copolymer shells significantly reduce adsorption of fibrinogen, HMWK, and FXII, thereby limiting both contact activation and platelet adhesion. The resulting coronas are sparse and enriched in albumin, a passivating protein that suppresses procoagulant signaling [65,66]. Consequently, aPTT is prolonged or unchanged (indicating minimal surface activation), the TG lag time increases, and TAT formation is minimal under shear [66,67,70,72,78,79].

Unlike PEGylation, which can suffer oxidative degradation and chain scission under long-term flow, zwitterionic coatings exhibit robust performance and chemical stability over months [70,78,79]. Importantly, these materials maintain hydration-mediated antifouling without blocking small-molecule diffusion, preserving gas and nutrient transport-key for tissue-engineered vascular grafts.

Zwitterionic grafting methods include surface-initiated polymerization, plasma deposition, and surface segregation from copolymers. In fibrous networks, conformal coverage is vital; discontinuous coatings permit local protein-binding “hot spots” that negate benefits. Achieving uniform deposition across high-curvature fibers requires precise control of surface energy and polymerization conditions [70,78,79].

### 5.4. Hybrid Fiber Strategies: Synergy Through Multi-Level Control

Single-mechanism methods are often unable to completely inhibit coagulation in complex physiological environments. Therefore, hybrid fiber systems combining heparin, NO donors, and zwitterion layers are emerging as the most effective solutions. The heparin + zwitterion combination combines enzyme inhibition with resistance to protein adsorption. The zwitterion coating reduces contact activation and platelet adhesion, while the underlying heparin layer neutralizes residual thrombin and factor Xa [68,70,78,79]. The NO + zwitterion combination allows for dual control of platelet activation and protein contamination. NO release maintains platelets in a resting state, while the zwitterion matrix minimizes donor consumption by limiting reactive adsorption sites [57,60,61,62]. The combination of heparin + NO + zwitterion creates a trivalent system that encompasses all three phases—initiation, amplification, and propagation—offering an efficiency almost mimicking the endothelium [68,70,74,77,78,79].

In preclinical models, such composites show significantly reduced thrombus mass, minimal complement activation (C3a and SC5b-9), and sustained hemocompatibility for >30 days under flow [66,74,76,77]. However, integrating these chemistries requires careful process design: ionic interactions between heparin and zwitterions can destabilize NO donors, while NO may oxidize thiol linkers in heparin layers. Advanced surface patterning and sequential polymerization mitigate such issues, ensuring chemical compatibility without compromising fiber mechanics [68,70,77,78,79]. To achieve comprehensive control over blood–material interactions, hybrid fiber systems combining multiple anticoagulant mechanisms have been developed. Table 4 outlines representative examples and their synergistic effects on coagulation kinetics and platelet activation.

### 5.5. Fiber Architecture and Morphology

Beyond chemistry, fiber morphology critically influences hemocompatibility. High-curvature submicron fibers amplify adsorption forces and can defeat even the best antifouling coatings if roughness exceeds 50–100 nm [65,70,78,79]. Thus, smooth, dense fiber bundles with reduced accessible area generally yield superior antithrombogenic performance.

Porosity controls plasma penetration: dense outer shells prevent internal fouling, while internal porosity supports mechanical flexibility and mass transport. For heparinized or zwitterionic fibers, ensuring uniform coating coverage across all accessible surfaces minimizes microdomain formation where coagulation could still initiate. Similarly, for NO-releasing materials, homogeneous donor distribution through fiber cross-sections supports continuous, low-flux release independent of external consumption [74,75,76].

Mechanical compliance also matters. Rigid fibers can amplify local shear gradients, enhancing platelet activation at interfaces; softer, hydrated materials mimic vascular elasticity, further reducing mechanosensory activation of circulating cells [70,78,79].

### 5.6. Regulation of Protein–Material Molecular Interactions: Initiation, Amplification, and Propagation Phases

Understanding how fiber-based materials influence blood coagulation requires a mechanistic rather than empirical approach. The interaction between polymeric surfaces and the coagulation cascade is highly multiscale: molecular interactions govern the formation of the initial protein corona, cellular responses drive platelet activation, and enzymatic feedback determines thrombin amplification. Within this continuum, anticoagulant and antithrombogenic fibers can be positioned according to how they modulate initiation, amplification, and propagation phases of the cell-based model of hemostasis [4,65,66].

At the onset of blood contact, protein adsorption acts as the decisive event. Unmodified hydrophobic fibers rapidly accumulate fibrinogen and HMWK, which undergo conformational rearrangement, exposing binding motifs that facilitate FXII autoactivation [65,66]. This process triggers the intrinsic (contact) pathway, resulting in spontaneous thrombin formation and subsequent platelet activation. In contrast, zwitterionic and highly hydrated fibers maintain a robust water layer that resists protein deposition and stabilizes adsorbed proteins in their native conformations [70,78,79]. This mechanism suppresses the contact pathway by preventing HMWK and FXII co-localization, yielding prolonged aPTT values and negligible FXIIa generation in plasma assays [66,67,72].

During the initiation phase, procoagulant fibers such as chitosan, oxidized cellulose, and calcium alginate promote the adsorption and conformational rearrangement of plasma proteins, including fibrinogen and HMWK, which facilitates autoactivation of FXII and kallikrein formation [73,74,75,76]. Positively charged composites based on chitosan and cationic PLA enhance this effect through electrostatic attraction, while alginate provides divalent ions that bind coagulation cofactors. These materials shorten the activated partial thromboplastin time (aPTT) and accelerate fibrin deposition [24,77,78,79,80]. Anticoagulant fibers counteract initiation by incorporating hydrated, zwitterionic, or pegylated coatings that prevent protein adsorption and preserve the native protein conformation [33,81,82]. A structured hydration layer on phosphorylcholine- or sulfobetaine-based surfaces reduces the entropic penalty for water displacement and prevents HMWK anchoring, thereby limiting factor XII binding. This mechanism results in prolonged aPTT and minimal contact activation, as evidenced by low plasma levels of factor XIIa and kallikrein and reduced complement activation [33,82,83,84,85].

During the amplification phase, early thrombin and ADP stimulate platelet activation, causing integrin clustering (GPIIb/IIIa) and phosphatidylserine (PS) exposure. At this stage, NO-releasing fibers act as dynamic regulators, maintaining platelet quiescence by enhancing intracellular cyclic GMP and reducing calcium influx [57,58,59]. Localized NO fluxes (10^−10–^10^−9^ mol cm^−2^ min^−1^) suppress P-selectin expression and block cytoskeletal reorganization, thereby preventing the assembly of intrinsic tenase and prothrombinase complexes. The result is delayed TG lag time, reduced peak thrombin, and low platelet adhesion even under physiologic shear conditions [50,67,68,74,75,76].

In the amplification phase, platelet activation and exposure to phosphatidylserine (PS) accelerate tenase and prothrombinase assembly [80,81]. Procoagulant fibers promote this process through surface roughness, microporosity, and local calcium enrichment, enhancing platelet adhesion and spreading [86,87,88]. In turn, NO-releasing coatings maintain platelets in a quiescent state by increasing cyclic GMP expression and reducing intracellular calcium, thereby inhibiting GPIIb/IIIa clustering and P-selectin exposure [89,90]. The resulting inhibition of platelet-driven propagation is reflected in reduced thrombin generation (TG) peaks and delayed lag times [24,89,90,91].

During propagation, procoagulant fibrils facilitate thrombin accumulation and fibrin polymerization, forming mechanically stable clots [86,87,88]. Heparinized fibrils, in contrast, recruit antithrombin III (ATIII), catalyzing the inactivation of FXa and FIIa to terminate thrombin bursts [92,93]. The efficiency of heparin functionalization depends on surface density, the availability of heparin chains, and their stability; optimized systems are characterized by low thrombin–antithrombin (TAT) complex formation and maintain fluid blood flow [94]. Taken together, these observations point to a continuous spectrum of material control, from positively charged, fibrinogen-binding surfaces that deliberately enhance coagulation to zwitterionic and heparinized architectures that suppress it [33,48,93].

Collectively, zwitterionic antifouling, NO-mediated platelet regulation, and heparin-based enzyme inhibition address distinct yet complementary stages of the coagulation cascade and allow blood-compatible fibrous materials to be tailored in a rational, mechanism-driven manner [49,51,79]. When combined, they emulate the functional triad of the vascular endothelium: hydration and charge neutrality (glycocalyx mimicry), continuous NO release, and localized heparan sulfate–ATIII anticoagulant activity.

However, multi-mechanistic designs introduce trade-offs. Overhydrated or highly zwitterionic surfaces may resist fouling but hinder endothelialization, crucial for tissue-integrated grafts. Conversely, high heparin densities can induce local bleeding or heparin-induced thrombocytopenia (HIT), while excessive NO release can produce oxidative by-products detrimental to cells [49,51,52,57,58,59]. The key challenge lies in quantitatively tuning surface functionality and maintaining spatial control of chemistry and flux within complex 3D fiber architectures.

### 5.7. Quantitative Mechanistic Assessment Indicators

To translate these materials into clinical applications, antithrombotic activity must be mechanistically quantified, linking molecular phenomena to functional coagulation endpoints. Conventional coagulation assays are only useful when interpreted in terms of pathway specificity and kinetic phase. aPTT (activated partial thromboplastin time) assesses the intrinsic/contact pathway. A prolonged aPTT indicates the suppression of factor XII/factor IX activation, characteristic of zwitterionic or NO-modified surfaces [49,50,55]. PT (prothrombin time) reflects the extrinsic pathway, which is essentially unaffected by surface chemistry and provides a benchmark for systemic coagulation competence [49]. TG (thrombin generation) measures dynamic clotting propagation—including lag time, peak thrombin concentration, and endogenous thrombin potential (ETP)—providing a quantitative fingerprint of the initiation, amplification, and propagation phases [49,50,55]. TAT complex concentration reflects cumulative thrombin exposure in flowing blood and serves as a sensitive indicator for comparative device evaluation under shear conditions [48,50,55]. Platelet activation markers (P-selectin, PAC-1 binding, and β-thromboglobulin release) correlate with amplification efficiency and indicate platelet-triggered activation pathways [48,50,57]. Complement activation (C3a and SC5b-9) and hemolysis index are crucial for long-term implants because complement-induced inflammation and erythrocyte lysis can accelerate thrombosis even in otherwise inert materials [48,49,50]. Together, these metrics form a coherent mechanistic testing matrix, linking each surface modification to its specific point of action in the coagulation cascade.

Quantitative coagulation metrics are essential for linking molecular interactions at the polymer–blood interface with macroscopic biological outcomes. Although empirical tests such as clotting time and bleeding index remain useful in preclinical assessment, they do not distinguish between the different phases of the coagulation cascade. Therefore, tests based on mechanistic analysis—specifically, activated partial thromboplastin time (aPTT), prothrombin time (PT), thrombin generation (TG), and quantification of the thrombin–antithrombin (TAT) complex—are essential for interpreting the effects of fibers on specific hemostasis checkpoints [24,91].

In procoagulant fibers, a shortened aPTT reflects factor XII activation via adsorption-induced conformational changes and enrichment of the contact phase complex (FXII/HMWK/kallikrein). This is typical for positively charged polysaccharide-based systems (e.g., chitosan and oxidized cellulose) and calcium-enriched alginate mats, where capillary action and ion exchange phenomena accelerate contact activation [24,69,70,71,72,73,74,75,76,77,78,79,80,81,82,83,84,85,86,87,88]. In contrast, anticoagulant fibers—especially those containing zwitterionic groups or dense pegylation—maintain prolonged aPTT values, indicating inhibited factor XII activation and minimized plasma protein adsorption [24,33,82,83,91]. Prothrombin time (PT), reflecting the tissue factor (extrinsic) pathway, remains largely unchanged for both classes of materials, confirming that the observed modulation is specific to the intrinsic/contact pathway [24,91].

The thrombin generation (TG) assay offers a dynamic picture of clotting kinetics, capturing the entire trajectory from initiation to propagation. Parameters such as lag time, peak thrombin concentration, and endogenous thrombin potential (ETP) serve as direct indicators of enzymatic feedback modulation between material surfaces [24,91]. Electrospun fibers based on chitosan or chitosan and PLA typically exhibit short lag times and elevated ETP, consistent with rapid tenase complex formation and strong platelet support [86,87,88]. In contrast, heparinized or NO-releasing surfaces exhibit delayed lag phases and lower peaks, indicating effective enzyme inhibition and platelet suppression [90,91,92,93].

Quantitative determination of TAT complexes provides a sensitive marker of cumulative thrombin exposure under flow conditions. Procoagulant mats, especially those rich in fibrin and Ca^2+^ crosslinks, are characterized by high TAT accumulation due to persistent thrombin activity on the surface [94]. Heparin-functionalized fibers drastically reduce TAT formation, confirming local ATIII recruitment and catalytic inactivation of FXa and FIIa [91,92,93,94].

Platelet activation assays (P-selectin, PAC-1, and β-thromboglobulin) complement these coagulation assays, revealing the involvement of cells in amplification. Chitosan- and rough PLA-based fibers induce marked platelet activation, while zwitterionic and NO-releasing coatings maintain platelets in a reversible quiescent state [63,85,92]. Markers of complement activation (C3a and SC5b-9/TCC) further expand this mechanistic profile by assessing inflammatory interactions; hydrophilic zwitterion layers demonstrate minimal complement activation compared to hydrophobic or unmodified controls [93]. Collectively, these assays form a mechanistic assessment matrix, allowing researchers to map specific material properties—charge, hydration, or catalytic activity—to their corresponding biological responses. Table 5 summarizes these correlations and highlights the characteristic biochemical signatures of procoagulant and anticoagulant fibers.

The data collectively indicate that thrombin generation and aPTT are the most sensitive markers for distinguishing procoagulant and anticoagulant mechanisms, while platelet and complement assays reveal a broader picture of biocompatibility [24,33,94].

### 5.8. Comparative Mechanisms and Translational Matrix

To provide a concise overview of how different material classes modulate distinct stages of the coagulation cascade, Table 6 summarizes representative fiber chemistries, their dominant mechanisms of action, and corresponding analytical readouts relevant to translational material design.

This matrix illustrates that no single material parameter ensures hemocompatibility; rather, hemocompatibility emerges from multi-level synergy between surface hydration, charge neutrality, catalytic inhibition, and controlled biochemical signaling.

From a translational standpoint, choosing an anticoagulant fiber architecture must balance mechanistic precision with clinical practicality. Short-term extracorporeal circuits and dialysis membranes benefit from covalently immobilized heparin due to strong propagation inhibition under high shear [73,76]. Long-term vascular implants, exposed to chronic blood flow, demand dual-modality surfaces: zwitterionic for persistent fouling resistance and NO-releasing for platelet control [77,78]. Tissue-engineered scaffolds require selective antifouling without impeding endothelial cell adhesion, achievable through micropatterned heparin-zwitterionic gradients that replicate endothelial heterogeneity [78,79].

From a mechanistic perspective, quantitative evaluation is essential for meaningful comparison of these systems and for guiding their further optimization. By correlating molecular design (charge, hydration, and functional groups) with biological metrics (aPTT, TG, TAT, platelet activation, and complement response), researchers can establish predictive relationships that guide fiber optimization for specific clinical roles [74]. The goal is not simply to create inert materials but to engineer hemocompatible interfaces that emulate the homeostatic behavior of living endothelium, capable of preventing thrombosis while preserving normal blood physiology.

The dynamic balance between coagulation activation and inhibition at the blood–material interface represents one of the most critical challenges in the design of fiber-based biomaterials. Both procoagulant and anticoagulant strategies aim to regulate the same physiological cascade but in opposite directions. The former accelerates clot formation to ensure rapid hemostasis in surgical or trauma settings, while the latter maintains long-term hemocompatibility in vascular grafts, catheters, and extracorporeal circuits [80,81]. Understanding how polymer chemistry, surface charge, hydration, and morphology collectively control protein adsorption, platelet signaling, and enzyme activity is key to designing functional fiber systems with predictable biological outcomes [33,85,86,87].

**Figure 5:**
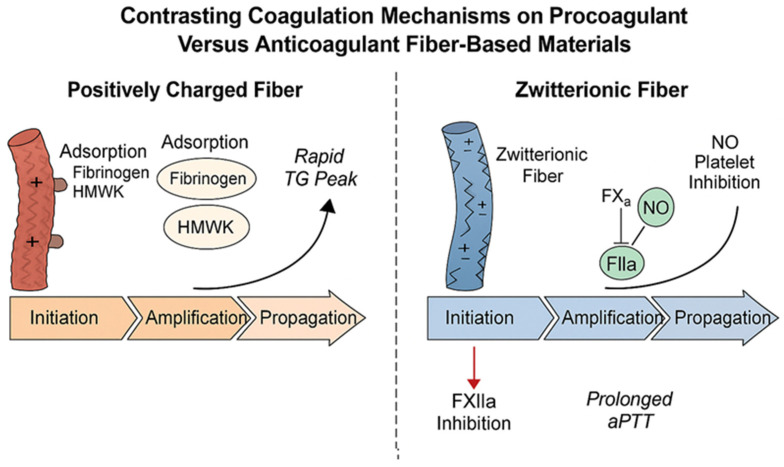
Contrasting coagulation mechanisms on procoagulant versus anticoagulant fiber-based materials. Positively charged and highly adsorptive fibers (e.g., chitosan, oxidized cellulose, Ca^2+^–alginate, and submicron PLA/PCL mats) promote fibrinogen and HMWK adsorption, accelerate FXII activation, and enhance platelet-driven amplification, resulting in a rapid thrombin generation peak [4,8,9,26,39,40,41,42,43,45]. In contrast, zwitterionic, heparin-functionalized, and NO-releasing fibers suppress protein adsorption, limit FXIIa formation, inhibit platelet activation, and prolong aPTT, collectively attenuating thrombin propagation under flow [48,49,75,79].

A visual comparison of these opposing mechanisms is provided in Figure 5, which integrates the experimentally established effects of procoagulant polysaccharides and synthetic fibers [4,8,9,26,50] with the antifouling, enzymatic, and platelet-suppressive behavior of zwitterionic, heparinized, and NO-releasing architectures [48,49,86,88].

### 5.9. Interfacial Chemistry and Morphological Drivers

Surface charge determines the first layer of interaction between blood and textile materials. Cationic fibers (e.g., chitosan) promote cell adhesion and coagulation by binding negatively charged membranes and proteins, while anionic polysaccharides (alginate and oxidized cellulose) support fibrin formation through Ca^2+^-mediated bridging and network stabilization [86,87,88,89]. Cationic polymers, such as chitosan (pKa ≈ 6.5), attract negatively charged erythrocytes and platelets, enhancing adhesion and clot initiation [69,70,71]. Mildly anionic materials, such as oxidized cellulose or alginate, can also stimulate factor XII activation but stabilize the fibrin network through calcium-mediated crosslinking [91].

Zwitterionic materials achieve charge neutrality and maintain a tightly bound hydration shell that resists protein adsorption below ~10 ng cm^−2^, especially when grafted as dense sulfobetaine or phosphorylcholine brushes [33,80]. These coatings mimic the glycocalyx of endothelial cells, balancing antifouling behavior with minimal inflammatory and complement activation [33,86].

Morphology and porosity further modulate coagulation. Electrospun procoagulant mats possess submicron fibers that create strong capillary forces, concentrating plasma proteins and promoting fibrin entrapment [90]. In contrast, anticoagulant coatings favor smooth, conformal morphologies that minimize curvature-induced protein unfolding and reduce the formation of a prothrombotic protein corona [33,48,65].

Mechanical stiffness also matters: stiffer mats increase platelet spreading and mechanotransduction, while soft, hydrated layers suppress integrin clustering, maintaining hemocompatibility [48,63,64,66]. This dual dependence on chemistry and mechanics is particularly evident in vascular grafts and dialyzer membranes, where long-term performance hinges on the simultaneous control of protein adsorption, platelet activation, and complement response [64,66,67,68].

Fiber diameter, porosity, and stiffness determine how blood permeates and interacts with the scaffold. Electrospun mats less than 500 nm in diameter accelerate clotting by trapping plasma proteins and platelets, while smoother and denser membranes delay activation [63,69,70,71]. Mechanically, procoagulant scaffolds benefit from stiffness that supports platelet spreading and fibrin anchoring, while compliant, hydrated coatings prevent mechanical triggering of integrin signaling and suppress thromboinflammatory feedback [48,63,64,66].

## 6. Logic and Perspectives of Functional Material Design

The literature supports a growing interest in hybrid systems that spatially integrate both mechanisms: for instance, vascular grafts with an anticoagulant inner lumen (zwitterion + heparin) and a prohemostatic outer sheath (chitosan + alginate) to aid tissue integration. These multilayer fibers mirror aspects of endothelial zonation, combining local bleeding control with global thrombosis prevention [64,65,66,67,68,69,70,71,72,73,92,93]. The concept underscores that hemocompatibility and hemostasis are not opposing goals but context-dependent expressions of the same surface physics (Table 7).

The comparative data confirm that the same fundamental physical parameters—charge, hydration, stiffness, and curvature—can be tuned bidirectionally to either amplify or suppress coagulation. While procoagulant materials exploit adsorption and mechanical feedback to reinforce hemostasis, anticoagulant systems use hydration, electrostatic neutrality, and catalytic inhibition to preserve blood fluidity [33,48,63,64,65,66,69,70,71,72,73,75,76]. The evolution of fiber-based biomaterials now moves toward multifunctional architectures that modulate these interactions spatially or temporally, allowing materials to behave like adaptive biological interfaces rather than static substrates [33,64,65,66,67,68,93].

This paradigm shift, supported by both experimental and theoretical studies on thrombin generation, protein adsorption, and complement activation [74,77,95], emphasizes predictive, mechanism-based design, a transition from empirical optimization to rational engineering of blood-compatible polymeric fibers.

Translating laboratory performance into clinical reliability requires multiscale integration of chemistry, morphology, and hemodynamics. For short-term topical applications, chitosan–alginate composites or oxidized cellulose remain highly effective due to their rapid fluid absorption, localized protein concentration, and intrinsic bioactivity [71,72,73,74,75,76,77,78]. For long-term blood-contacting devices, the challenge is sustained hemocompatibility under shear and biofouling stress.

Hybrid coatings that combine zwitterionic hydration with NO-mediated platelet regulation or heparin-catalyzed enzyme inhibition represent the most promising strategies [73,85,93]. Tri-modal surfaces have already shown strong reductions in platelet adhesion and TAT formation while maintaining stable mechanical and chemical performance in simulated circulation [81,82,96].

The future direction lies in adaptive and biomimetic fiber systems capable of switching between pro- and anticoagulant states. Stimuli-responsive coatings that alter charge or hydration in response to pH, redox potential, or ionic strength could allow on-demand modulation of hemostasis, similar to endothelial homeostasis [89,91]. Integrating machine learning-based optimization with high-throughput hemocompatibility screening may further accelerate the design of next-generation fibers that are no longer inert but actively regulate the coagulation environment [80,85,91,94]. Ultimately, both strategies—procoagulant and anticoagulant—represent complementary facets of material–blood interaction. Their convergence in hybrid systems illustrates a paradigm shift: from static coatings to dynamic, feedback-controlled interfaces that emulate the adaptability of the vascular endothelium [90,92,93].

### The Clinical Implementation of Hemostatic Biomaterials

The clinical implementation of hemostatic biomaterials is associated with stringent requirements, where biological safety must be combined with a technologically reproducible production process. Regulatory authorities classify most advanced hemostatic materials as Class III medical devices (high risk) [96]. The MDR) introduced the requirement to present hard clinical data for each device. A key challenge is demonstrating clinical benefits while avoiding adverse effects such as embolism or excessive inflammatory response: EMA Quality Guidelines [96,97]. The FDA (PMA—Premarket Approval) requires rigorous biocompatibility testing according to the ISO standards, with particular emphasis on hemocompatibility testing. A new trend is the requirement to test materials in flow (dynamic) models, not just static ones. The issue of “borderline products” is often a challenge during the implementation process, as hemostatic materials often straddle the line between a medical device and a medicinal product (e.g., when containing thrombin or growth factors), complicating the certification process [96,97,98].

Sterilization is a critical step that often degrades the unique properties of polymers. Irradiation (gamma/E-beam) can cause crosslinking or degradation of polymer chains, altering the mechanical properties of membranes or the swelling rate of hydrogels. This can lead to a loss of prohemostatic properties [96,98]. Ethylene oxide (EtO) is the standard for heat-sensitive materials, but the challenge lies in completely removing residual gas from the porous fibrillar structures, which is monitored by stringent toxicity limits. Modern methods such as plasma sterilization (hydrogen peroxide) are gaining popularity, but they can alter the surface energy of the material, affecting platelet adhesion [97,99].

The transition from laboratory to large-scale production also encounters technological barriers. Large-scale production of materials with fibrillar geometry via electrospinning is challenging due to the need to maintain identical fiber diameter and porosity over large surfaces [100]. Multi-nozzle or needleless electrospinning systems offer a solution. Maintaining stable environmental parameters (process control) is essential for reproducible morphology of flat and porous surfaces (e.g., humidity and temperature control) [98,99].

In biomaterials production, ensuring consistent performance from batch to batch (batch consistency) is crucial. Natural polymers (e.g., chitosan and collagen) exhibit significant biological variability. The shift to synthetic polymers or recombinant proteins with precisely defined molecular weights is becoming the norm [100,101]. Manufacturers must monitor critical quality parameters (CQAs), including pore size distribution, surface functional group density, and active ingredient release kinetics. Analytical verification is also essential, using analytical methods such as SEM microscopy with AI image analysis to automatically monitor fiber geometry in each production batch [96,101]. Currently, the market success of hemostatic biomaterials depends on design for manufacturing. The best fibrillar geometry will not find clinical use unless its manufacturing process is resistant to sterilization and can be validated in accordance with FDA Quality System Regulation guidelines. This is, therefore, one of the greatest challenges facing contemporary medical materials engineering for the future implementation of innovative solutions in this field [97,98,100].

## 7. Conclusions

Fiber-forming polymers provide an exceptionally versatile platform for engineering interactions between synthetic materials and the human coagulation system. Their high surface area, tunable charge, and controlled porosity enable precise manipulation of plasma protein adsorption, platelet signaling, and enzymatic activity, allowing the same structural motif, a fiber, to function as either a hemostatic accelerator or a hemocompatible interface, depending on its chemistry and morphology [4,48,102,103]. The findings reviewed herein demonstrate that coagulation is not simply triggered or inhibited by a material’s bulk composition but is dynamically governed by the nature of the protein corona and the subsequent cellular and enzymatic events occurring on the fiber surface [48,102,103].

In procoagulant systems, fibers composed of chitosan, alginate, or oxidized cellulose act as active scaffolds for localized clot formation. Their charged and hydrophilic domains promote adsorption of fibrinogen and high-molecular-weight kininogen (HMWK), facilitate the autoactivation of FXII, and support the intrinsic coagulation cascade. The combination of positive surface potential, calcium release, and capillary wicking accelerates platelet aggregation and fibrin network formation, leading to shortened activated partial thromboplastin time (aPTT), higher thrombin generation peaks, and rapid hemostatic performance in vivo [8,80,104,105,106,107]. The clinical relevance of these materials has been confirmed in surgical dressings and trauma pads based on chitosan and oxidized regenerated cellulose, where rapid and localized hemostasis is required without systemic activation [8,87,105,106].

In contrast, anticoagulant and antithrombogenic fibers aim to maintain blood fluidity during long-term contact. Their performance relies on hydration mediated antifouling, localized enzyme inhibition, and mimicry of endothelial signaling. Zwitterionic coatings such as phosphorylcholine- and sulfobetaine-based polymers maintain strong hydration shells that suppress protein adsorption and prevent FXII/HMWK complexation, effectively prolonging aPTT and minimizing contact activation [33,108,109]. Nitric oxide-releasing surfaces complement this effect by maintaining platelet quiescence through cyclic GMP signaling, reducing P-selectin exposure and phosphatidylserine translocation [87,88,89]. Meanwhile, heparin-functionalized fibers and electrospun vascular grafts provide catalytic inhibition of FXa and thrombin (FIIa) via antithrombin III (ATIII) recruitment, resulting in lower thrombin–antithrombin (TAT) levels and depressed endogenous thrombin potential (ETP) in plasma and whole-blood assays [110,111].

These mechanisms highlight how material–protein–cell interactions can be directed toward opposite ends of the hemostatic spectrum by adjusting only interfacial parameters such as charge density, hydration, and topography. Theoretical and experimental frameworks, including the cell-based model of coagulation and calibrated thrombin generation assays, have provided mechanistic insight into how these parameters map onto the three functional phases of coagulation: initiation, amplification, and propagation [4,24,48,102,105,110]. The growing consensus across the literature is that designing hemocompatible materials requires a shift from empirical observation to mechanistic under-standing, where performance is predicted from quantitative correlations between surface properties and pathway-specific assays such as aPTT, thrombin generation, and TAT formation [24,112,113].

Future research directions are converging toward hybrid, adaptive, and hierarchical fiber systems capable of dynamic regulation of blood responses. Hybrid architectures combining zwitterionic hydration with nitric oxide release and heparin-mediated enzyme inhibition have demonstrated synergistic reduction in both platelet activation and thrombin generation under physiologic shear [33,108,109,110,111,112]. Spatially graded or coaxial electrospun fibers could enable dual-function materials, where the luminal surface remains antithrombogenic while the external sheath promotes tissue integration or localized hemostasis [8,89,114,115]. Beyond static coatings, the next generation of materials will likely employ stimuli-responsive chemistries that alter hydration or charge in response to pH, ionic strength, or oxidative stress, enabling self-regulated transitions between hemostatic and anticoagulant modes [33,108,109,110,111,112].

Another emerging direction involves data-driven design and multiscale modeling, integrating descriptors such as zeta potential, roughness, and surface hydration energy with experimental coagulation metrics (aPTT, TG, TAT, and platelet activation) to establish predictive, quantitative structure–biocompatibility relationships. Such approaches will accelerate the discovery of new fiber chemistries optimized for specific clinical contexts—ranging from topical hemostats to vascular graft coatings—while reducing experimental redundancy [4,24,48,102,114].

Finally, the translational success of these systems will depend on standardization of hemocompatibility testing and validation under physiologic flow. Combining static and dynamic assays (e.g., TG, aPTT, complement activation, and platelet adhesion) within reproducible flow models will enable cross-study comparison and establish mechanistic benchmarks for regulatory approval [24,48,114,115].

In conclusion, fiber-based polymeric materials represent an evolving class of smart interfaces that can either promote or inhibit coagulation by rational manipulation of surface physics and chemistry. Their future lies in integrative designs that mimic endothelial adaptability-balancing hemostasis and antithrombosis through self-regulating mechanisms. As mechanistic understanding deepens and quantitative frameworks mature, the boundary between hemostatic biomaterials and hemocompatible devices will blur, giving rise to a new generation of adaptive, precision-engineered fibers that respond intelligently to the dynamic demands of the circulatory system [4,8,24,33,112,113,114,115].

## Figures and Tables

**Table 1:** Summary of polymer physicochemical parameters influencing blood coagulation pathways.

Polymer Property	Mechanistic Effect	Biological Consequence	Ref.
Positive surface charge (e.g., chitosan)	Attracts fibrinogen/HMWK; promotes FXII binding	Shortened aPTT; enhanced thrombin generation	[4,9,27]
Negative charge (e.g., alginate and ORC)	Ca^2+^ binding; moderate FXII activation	Rapid fibrin polymerization	[26,39]
High surface curvature	Induces fibrinogen unfolding; platelet clustering	Accelerated platelet activation	[33,40]
Hydration/zwitterionic shell	Prevents protein adsorption	Prolonged aPTT; reduced platelet adhesion	[8,16]
Soft mechanical compliance	Limits platelet spreading	Reduced PS exposure	[23]

**Table 2:** Chemical structure–function relationship in representative fiber-forming polymers.

Polymer	Dominant Functional Group(s)	Typical Surface Charge (pH 7.4)	Biological Effect on Coagulation	Ref.
**Chitosan**	-NH_2_ (primary amine, protonated)	Positive (cationic)	Promotes platelet adhesion and activation; shortens aPTT; enhances fibrin formation via FXII/HMWK binding	[4,8,9,27,50,51]
**Alginate**	-COO^−^ (carboxylate, Ca^2+^-crosslinked)	Negative (anionic)	Releases Ca^2+^; accelerates FX activation and fibrin polymerization; may trigger mild contact activation	[26,27,52,53]
**Oxidized cellulose (ORC)**	-COOH (oxidized hydroxyl groups)	Negative (anionic)	Concentrates plasma and coagulation proteins; promotes fibrin network anchoring; mild FXII activation	[4,26,39,54]
**PLA/PCL fibers**	-COOR (ester), hydrophobic backbone	Neutral/slightly negative	Promotes fibrinogen unfolding and platelet adhesion on curved submicron fibers; enhances thrombin generation	[4,50,55]
**Heparin**	-SO_3_^−^, -COO^−^ (sulfated polysaccharide)	Strongly negative	Binds antithrombin III; inhibits FXa and FIIa; prolongs aPTT; suppresses thrombin generation	[4,55,56,57]
**Zwitterionic polymers (PC, SB, and CB)**	-N^+^(CH_3_)_3_/-SO_3_^−^ or -COO^−^ (dipolar groups)	Net neutral	Forms stable hydration layer; suppresses protein adsorption, FXII/HMWK recruitment, and platelet activation	[8,16,57,58]

**Table 3:** Comparison of natural and synthetic polymers in key biomedical aspects.

Characteristic	Natural Polymers	Synthetic Polymers	Ref.
**Mechanical Properties**	Typically weak; low tensile strength and brittleness	High and configurable; high strength and flexibility	[58,59]
**Degradation**	Enzymatic, rapid, and often difficult to precisely control	Hydrolytic, predictable, and controllable (from weeks to years)	[59,60]
**Hydration (Hydrophilicity)**	Usually high (form hydrogels); excellent water binding	Variable (often hydrophobic) but modifiable	[58,60,61]
**Rate of Hemostasis**	Very high; possess natural protein and platelet-binding motifs (e.g., RGD)	Low; function primarily mechanically unless chemically modified	[59,61,62]
**Cost**	Often lower (renewable raw materials) but with high medical purification costs	High synthesis and certification costs for chemical processes	[62,63]
**Clinical Readiness**	High (widely used in dressings and natural threads)	High for specific groups (e.g., FDA-approved PLA/PGA threads)	[61,63,64]

**Table 4:** Representative hybrid fiber systems and their combined anticoagulant mechanisms.

Hybrid Composition	Main Components/Coatings	Mechanistic Synergy	Observed Effect (aPTT/TG/Platelet)	Application	Ref.
Heparin + Zwitterion	Heparinized base + phosphorylcholine brush	ATIII-mediated enzyme inhibition + antifouling hydration	Prolonged aPTT and ↓ TG peak	Vascular grafts	[68,70,78]
NO + Zwitterion	S-nitrosothiol donor in sulfobetaine matrix	Platelet suppression + protein resistance	↓ P-selectin and delayed TG lag	Catheters and stents	[74,76,77]
Heparin + NO	Covalently coupled layers	Enzymatic inhibition + platelet quiescence	↓ TAT and prolonged TG lag	Dialysis membranes	[71,74,75]
Heparin + NO + Zwitterion	Multilayer copolymer	Full control of initiation/amplification/ propagation	Endothelium-like hemocompatibility	Long-term implants	[68,74,77,78,79]

**Table 5:** Mechanistic interpretation of standard coagulation metrics for fiber-based materials.

Coagulation Metric	Pathway/ Mechanistic Target	Procoagulant Behavior	Anticoagulant Behavior	[Ref.]
aPTT (activated partial thromboplastin time)	Intrinsic/contact activation (FXII/HMWK)	Shortened due to enhanced FXII autoactivation and contact factor binding	Prolonged; suppressed FXII binding and reduced HMWK interaction	[33,85,95]
PT (prothrombin time)	Extrinsic pathway (tissue factor)	Largely unchanged; minimal sensitivity to contact-driven changes	Largely unchanged	[24,91]
TG (thrombin generation)	Global coagulation kinetics (initiation → propagation)	High peak thrombin and ETP; short lag time	Low peak; delayed lag; reduced ETP	[24,93]
TAT complex	Cumulative thrombin exposure in vivo	Elevated (sustained thrombin activity)	Reduced (ATIII-mediated FXa/FIIa inhibition)	[92,93,94]
Platelet activation (P-selectin and PAC-1)	Amplification/ PS exposure	Strong upregulation; largely irreversible activation	Minimal; reversible adhesion only	[86,87,88,89,90]
Complement activation (C3a and SC5b-9)	Inflammatory cross-talk/ foreign surface recognition	Moderate to high	Negligible to low	[33,85]

**Table 6:** Comparative mechanisms and translational framework for fiber-based anticoagulant materials.

Mechanistic Target	Representative Fiber Chemistry	Dominant Mode of Action	Coagulation Phase Affected	Key Analytical Metric(s)	Typical Application	Ref.
**Contact** **activation** **inhibition**	Zwitterionic (PC, SB, and CB), PEGylated coatings	Hydration-layer formation; suppression of FXII/HMWK adsorption and activation	*Initiation*	aPTT, FXIIa assays, and protein adsorption studies	Vascular grafts and catheters	[70,78,79]
**Platelet** **activation control**	Nitric oxide (NO)-releasing fibers	GPIIb/IIIa suppression, reduced PS exposure, inhibition of platelet aggregation	*Amplification*	TG lag time, P-selectin, and PAC-1 binding	Catheters and stents	[74]
**Enzymatic propagation suppression**	Heparin-functionalized fibers	Antithrombin III-mediated inhibition of FXa/FIIa	*Propagation*	TG (peak/ETP), TAT complex, and aPTT	Dialysis membranes and extracorporeal circuits	[68,71]
**Multi-phase control** **(synergistic)**	Hybrid NO–Heparin–Zwitterion fibers	Synergistic antifouling, platelet quiescence, and enzyme inhibition	*All phases*	aPTT, TG, TAT, and platelet and complement assays	Long-term implants and artificial organs	[68,72,73,74,75,76,77]

**Table 7:** Comparative material–mechanism matrix for procoagulant and anticoagulant fibers.

Design Parameter	Procoagulant Fibers	Anticoagulant/Antithrombogenic Fibers	[Ref.]
Representative Polymers	Chitosan, alginate, oxidized cellulose, and PLA/PCL composites	Heparinized polymers, zwitterionic copolymers, and NO-releasing systems	[33,65,96]
Dominant Surface Charge	Positive or moderately negative	Neutral or zwitterionic	[33,65,95]
Hydration and Protein Adsorption	Limited hydration; high fibrinogen/HMWK adsorption	Strong hydration; low adsorption (<10 ng cm^−2^)	[33,65,87]
Protein Corona Composition	Fibrinogen, HMWK, and FXII rich	Albumin dominated; native conformations	[33,48,91,92,93,94,95]
Platelet Interaction	Strong adhesion; PS exposure; aggregation ↑	Weak, reversible adhesion; quiescent phenotype	[63,64,69,85]
Thrombin Activity	Accelerated generation; high ETP	ATIII-mediated inhibition of FXa/FIIa; low TAT	[72,92,93,94,95]
Mechanical Compliance	Stiff, porous matrices promoting clot anchoring	Soft, hydrated coatings preventing mechanotransduction	[48,63,78]
Typical Application	Topical hemostats, trauma pads, and surgical dressings	Catheters, vascular grafts, dialyzers, and long-term implants	[48,63,64,66,67,68,69,70,71,72,73,74,75,76,77,78,79,80,81,82,83,84,85]
Dominant Biological Outcome	Rapid fibrin formation and localized bleeding control	Sustained hemocompatibility and thrombosis prevention	[33,68,72,86]

## Data Availability

No new data were created or analyzed in this study.

## Abbreviations

The following abbreviations are used in this manuscript:

ADP	Adenosine Diphosphate
anti-Xa	Heparin Assay
aPTT	Kaolin–Cephalin Time
ATIII	Antithrombin III
CB	Carboxybetaine
ETP	Endogenous Thrombin Potential
GPIIb	Glycoprotein Receptor Inhibitor
H12	γ-chain Dodecapeptide
HIT	Heparin-induced Thrombocytopenia
HMWK	High-Molecular-Weight Kininogen
IgG	Immunoglobulin G
LbL	Layer by Layer
ORC	Oxidized Regenerated Cellulose
PAC-1	Procaspase-3 Activator
PC	Phosphorylcholine
PCL	Polycaprolactone
PEG	Polyethylene Glycol
PF4	Platelet Factor 4
PK	Prekallikrein
PLA	Poly(lactic acid)
PS	Phosphatidylserine
PT	Prothrombin Time
PU	Polyurethane
SB	Sulfobetaine
SEM	Scanning Electron Microscopy
TAFI	Thrombin Fibrinolysis Inhibitor
TAT	Thrombin–Antithrombin III
TF	Tissue Factor
TG	Thrombin Generation
